# Supplementary material for: Space-by-Time Modular Decomposition Effectively Describes Whole-Body Muscle Activity During Upright Reaching in Various Directions
Source: Front Comput Neurosci. 2018 Apr 3;12:20. doi: 10.3389/fncom.2018.00020 (PMC5891645; doi:10.3389/fncom.2018.00020)
Supplement: Supplementary file 1 [file Image1.PDF]

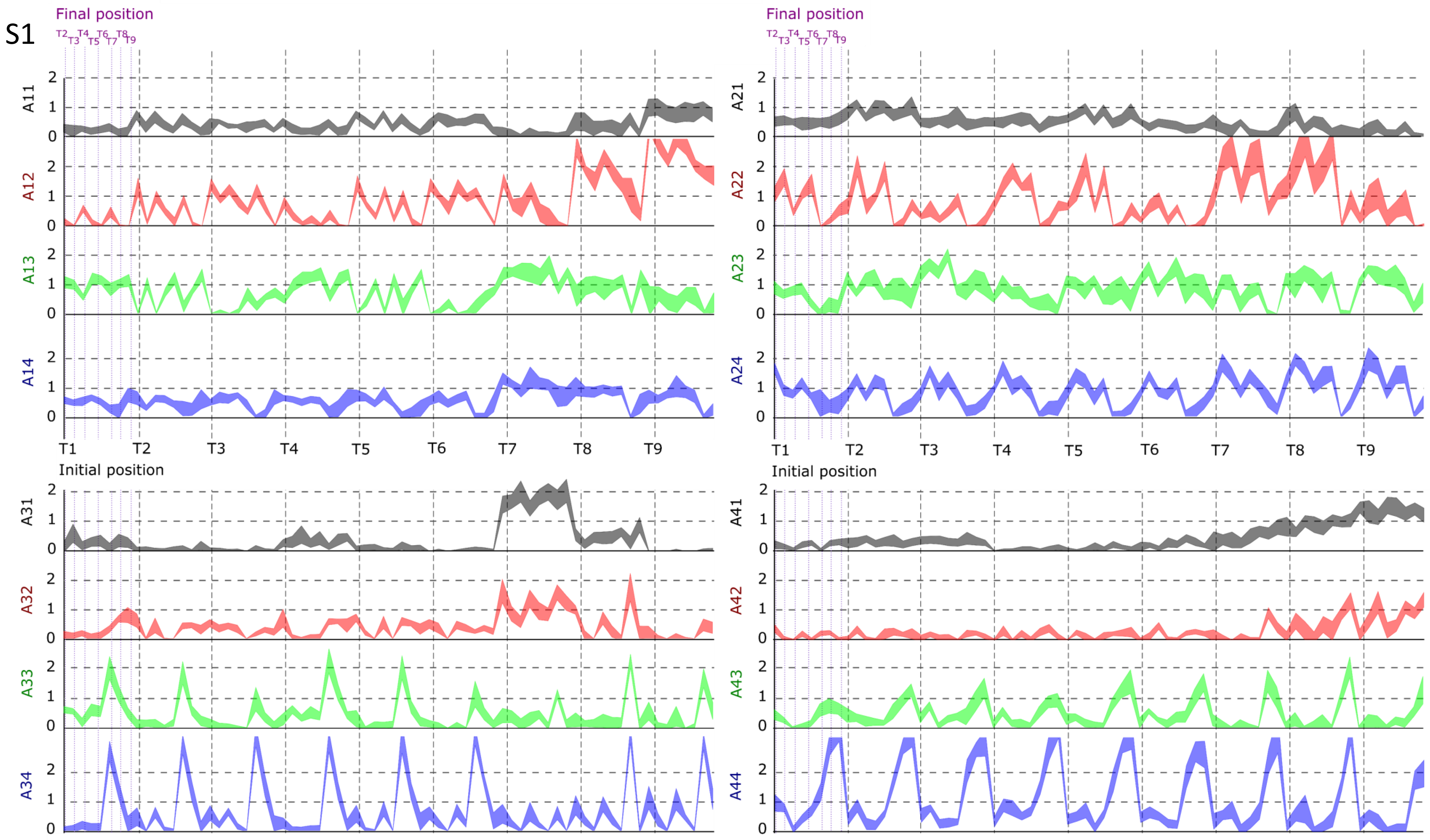

Figure A.1: Decoding coefficient in function of initial and final posture for the subject 1. Each graph represent the value of an a_{ij} coefficient in function of the initial position (from T1 to T9; grey dotted vertical bars) and the final position (from T1 to T9; violet dotted vertical bars), with i being the number associated to temporal modules (from 1 to 4) and j to spatial modules (from 1 to 4). Each pannel represents one temporal module (1-left up, 2-right up, 3-left down, 4-right down), each color one spatial module (1-grey, 2-red, 3-green, 4-blue).

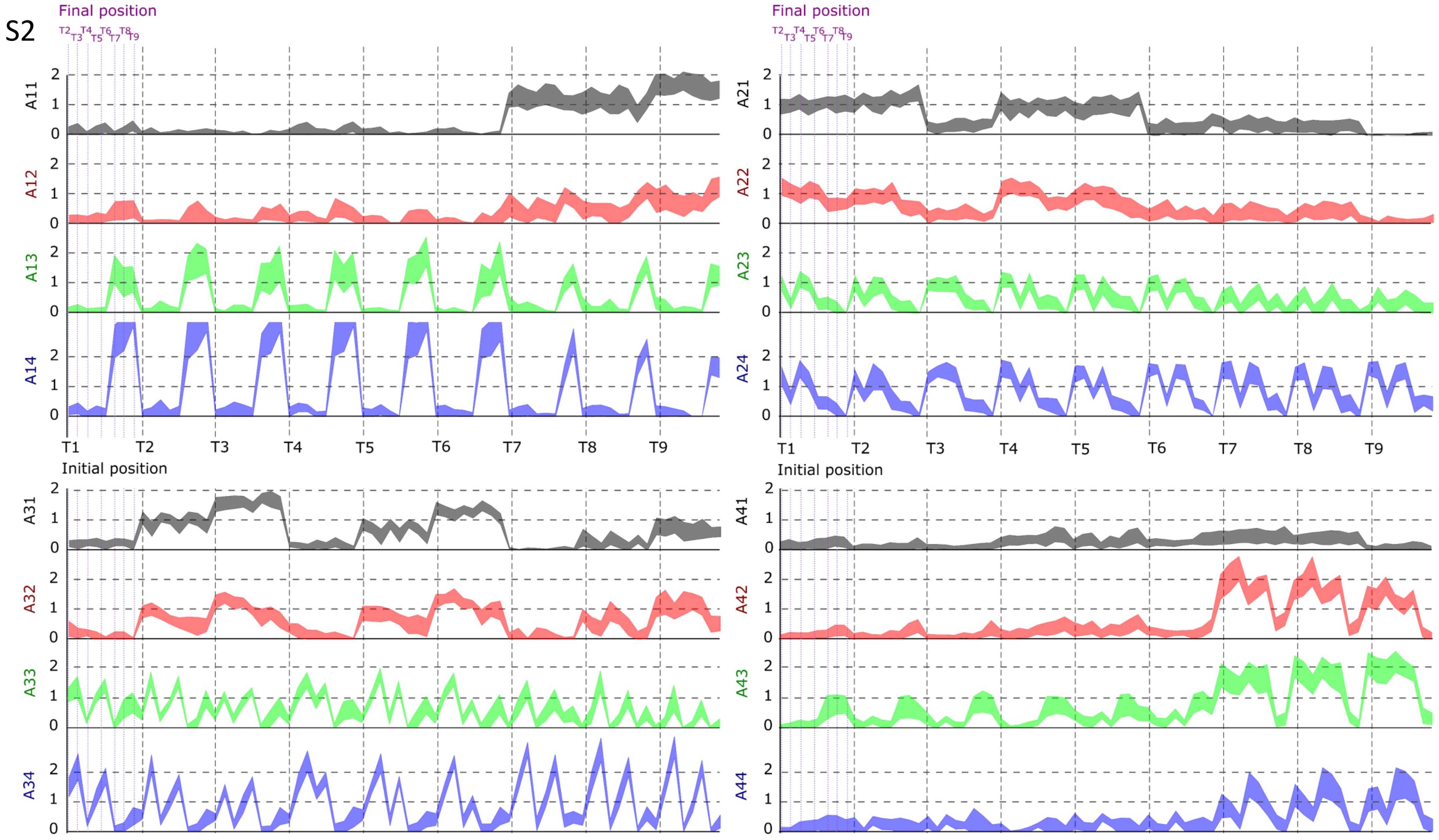

Figure A.2: Decoding coefficient in function of initial and final posture for the subject 2. Each graph represent the value of an a_{ij} coefficient in function of the initial position (from T1 to T9; grey dotted vertical bars) and the final position (from T1 to T9; violet dotted vertical bars), with i being the number associated to temporal modules (from 1 to 4) and j to spatial modules (from 1 to 4). Each pannel represents one temporal module (1-left up, 2-right up, 3-left down, 4-right down), each color one spatial module (1-grey, 2-red, 3-green, 4-blue).

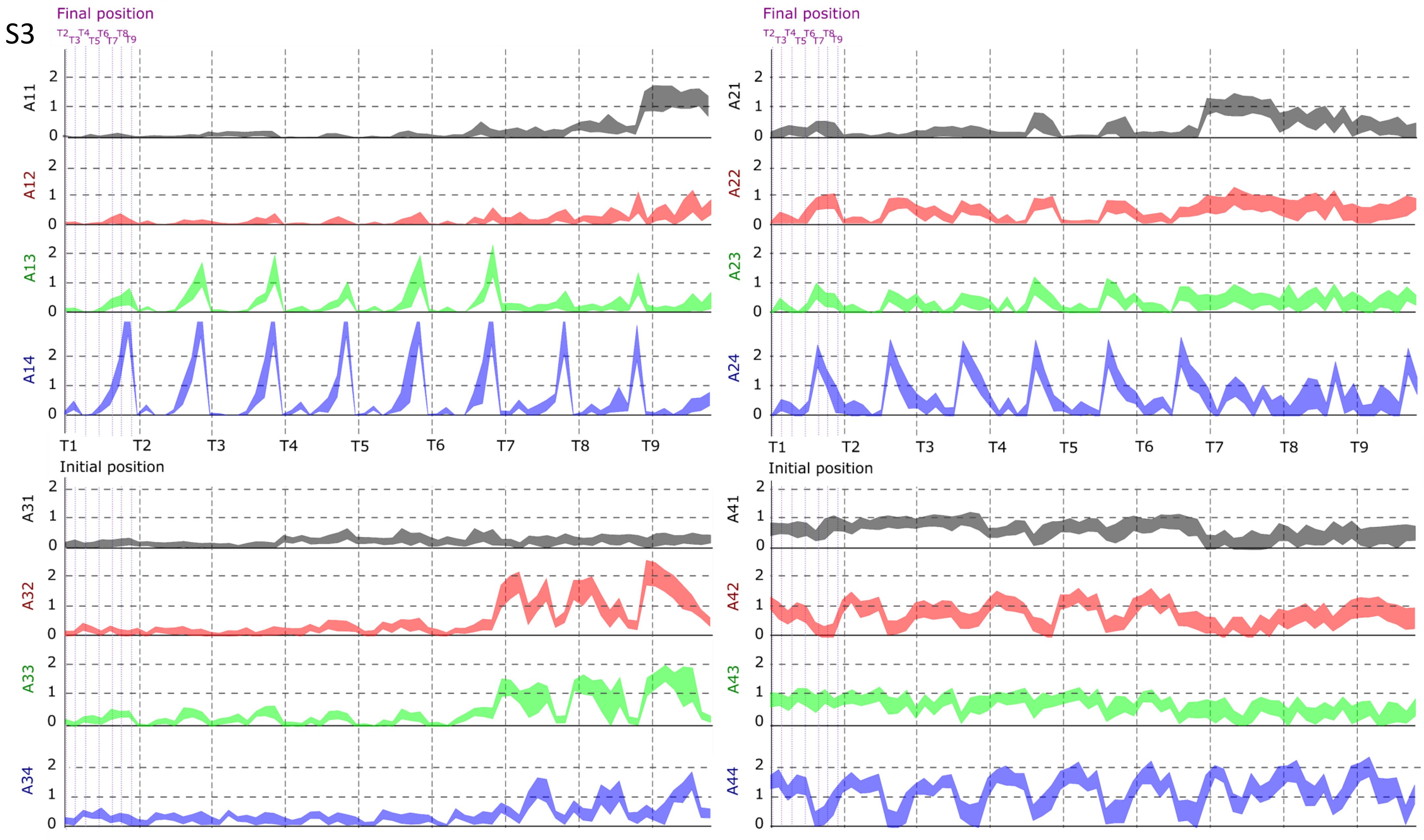

Figure A.3: Decoding coefficient in function of initial and final posture for the subject 3. Each graph represent the value of an a_{ij} coefficient in function of the initial position (from T1 to T9; grey dotted vertical bars) and the final position (from T1 to T9; violet dotted vertical bars), with i being the number associated to temporal modules (from 1 to 4) and j to spatial modules (from 1 to 4). Each pannel represents one temporal module (1-left up, 2-right up, 3-left down, 4-right down), each color one spatial module (1-grey, 2-red, 3-green, 4-blue).

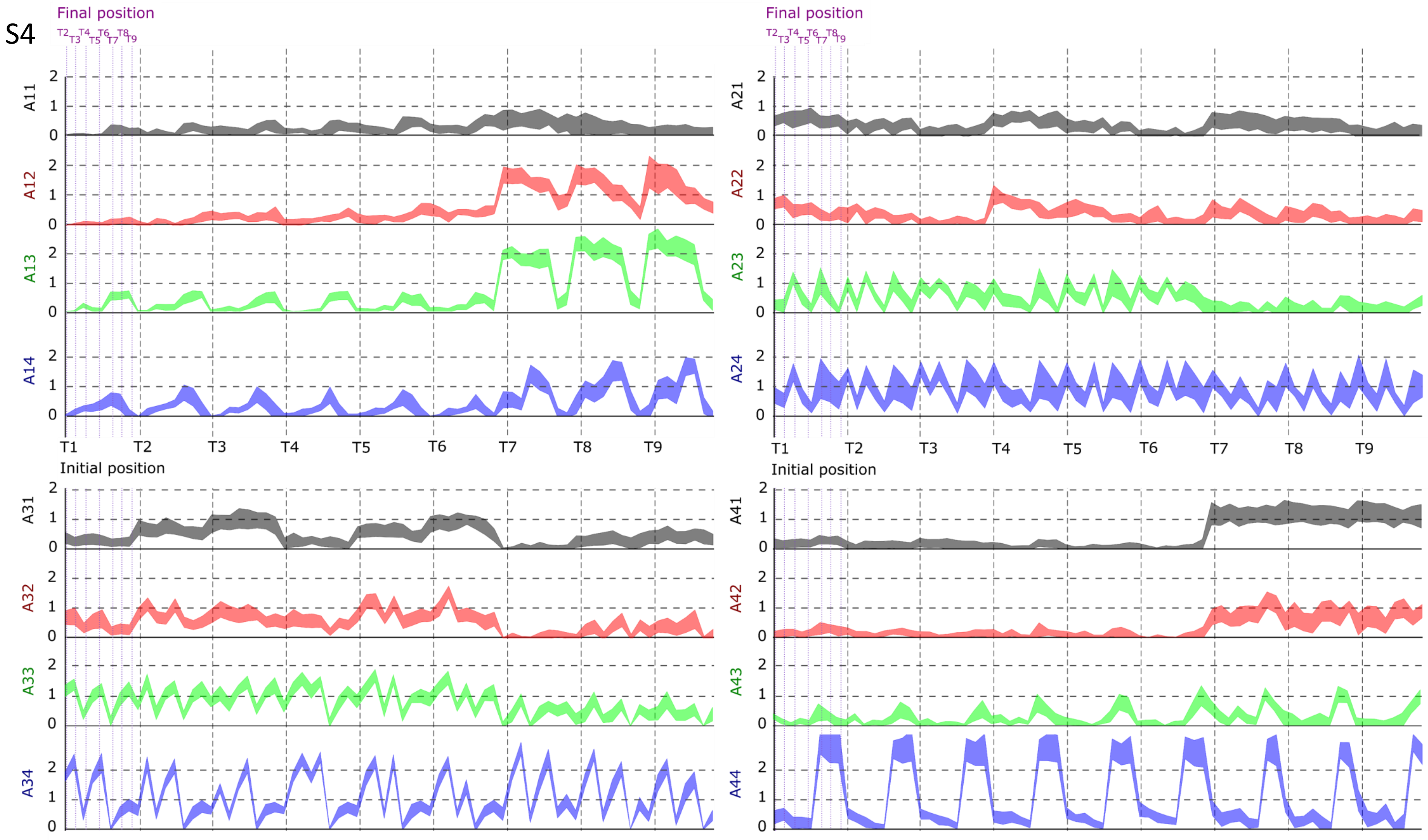

Figure A.4: Decoding coefficient in function of initial and final posture for the subject 4. Each graph represent the value of an a_{ij} coefficient in function of the initial position (from T1 to T9; grey dotted vertical bars) and the final position (from T1 to T9; violet dotted vertical bars), with i being the number associated to temporal modules (from 1 to 4) and j to spatial modules (from 1 to 4). Each pannel represents one temporal module (1-left up, 2-right up, 3-left down, 4-right down), each color one spatial module (1-grey, 2-red, 3-green, 4-blue).
